# A new diagnostic work-up for defining anemia etiologies: a cohort study in patients ≥ 50 years in general practices

**DOI:** 10.1186/s12875-020-01241-7

**Published:** 2020-08-16

**Authors:** A. Schop, K. Stouten, J. A. Riedl, R. J. van Houten, M. J. G. Leening, J. van Rosmalen, P. J. E. Bindels, M-D Levin

**Affiliations:** 1 Department of Internal Medicine, Albert Schweitzer Hospital, Postbus 444, 3300 AK Dordrecht, the Netherlands; 2 Department of Clinical Chemistry, Albert Schweitzer Hospital, Dordrecht, the Netherlands; 3 General practice van Houten, Hendrik-Ido-Ambacht, the Netherlands; 4 Departments of Epidemiology and Cardiology, Erasmus MC – University Medical Center Rotterdam, Rotterdam, the Netherlands; 5 Department of Biostatistics, Erasmus MC, Rotterdam, the Netherlands; 6 Department of General Practice, Erasmus MC – University Medical Center Rotterdam, Rotterdam, the Netherlands

**Keywords:** Hematologic diseases, General population, Diagnostic work-up, Public health

## Abstract

**Background:**

To study etiologies of anemia using an extensive laboratory analysis in general practices.

**Method:**

An extensive laboratory analysis was performed in blood of newly diagnosed anemia patients aged ≥50 years from the general population in the city of Dordrecht area, the Netherlands. Eight laboratory-orientated etiologies of anemia were defined. Patients were assigned one or more of these etiologies on the basis of their test results.

**Results:**

Blood of 4152 patients (median age 75 years; 49% male) was analyzed. The anemia etiology was unclear in 20%; a single etiology was established in 59%; and multiple etiologies in 22% of the patients. The most common etiologies were anemia of chronic disease (ACD) (54.5%), iron deficiency anemia (IDA) (19.1%) and renal anemia (13.8%). The most common single etiologies were IDA (82%) and ACD (68%), while the multiple etiologies most commonly included folic acid deficiency (94%) and suspected bone marrow disease (88%). Older age was associated with a lower incidence of IDA and a higher incidence of renal anemia. Mild anemia was more often associated with ACD and uncertain anemia, while severe anemia was mainly seen in patients with IDA.

**Conclusion:**

Extensive laboratory analysis in anemic patients from the general population helped clarify the etiology of anemia and revealed many various combinations of etiologies in a significant proportion of patients. Age, sex and the severity of anemia are predictive of the underlying etiology.

## Background

In clinical practice, anemia diagnostics is a four-step process, starting with the clinical suspicion of anemia and confirmation of a decreased hemoglobin concentration in a blood test. Secondly, the anemia can be further categorized into an underlying etiology based on additional laboratory analysis. The third step is to match the anemia etiology to the patient’s clinical presentation, symptoms and comorbidities. If necessary, additional diagnostic tests can be performed (such as endoscopy) to find the cause of the anemia. The final step is to confirm the underlying condition that is causing anemia and to treat this to prevent relapse of the anemia or a decline in clinical condition [1, 2].

In the Western world, four common etiologies for anemia are anemia of chronic disease (ACD) (17–40%), iron deficiency anemia (IDA) (5–19%), renal anemia (2–15%) and vitamin B12 or folic acid deficiency (2–14%) [3–6]. For still 26–44% of patients, the etiology of anemia is uncertain. Anemia in relation to aging and sex has been studied previously, although but few studies distinguished between the various anemia etiologies [2, 3]. One study in a cohort of anemic women aged ≥65 years found a higher incidence of ACD and renal anemia in older age [4]. Another study in a general population found an increasing incidence of ACD and uncertain anemia etiology with higher age, while IDA was more common in females [5].

Studies concerning anemia etiologies in community-dwelling patients usually present data obtained from either a limited or a step-wise laboratory analysis. In one study, the use of a limited laboratory analysis was compared to a more extensive laboratory analysis. They found that an extensive laboratory analysis leads more often to establishment of the anemia etiology [7]. Moreover, extensive laboratory analysis would prevent a delay in the initiation of treatment for the underlying disease and has shown to be cost-effective [8]. Another drawback of a limited or step-wise laboratory analysis is that this cannot assess the multifactorial role of etiologies causing anemia. In some previous studies, multiple causes of anemia were found in about 30–50% of included patients [9, 10].

We set out to systematically study common etiologies of anemia and the role of multiple etiologies of anemia on the basis of the results of extensive laboratory analysis in a population of newly diagnosed patients in general practices. Furthermore, we evaluated the severity of anemia and its relation with the underlying etiology or etiologies.

## Methods

### Study population

A cohort study was designed for our research purpose. Patient data were collected from a large database set up in the laboratory of the Albert Schweitzer Hospital, Dordrecht, the Netherlands. This project was of multidisciplinary origin; general practitioners (GPs), internal medicine physicians and clinical chemists participated. Eighty-one of the 150 GPs in the city of Dordrecht area, the Netherlands, agreed to participate in this project, which started on 1 February 2007. The department of clinical chemistry of the Albert Schweitzer Hospital is the referral laboratory for GPs in the area.

If a participating GP requested a blood test for a patient aged ≥50 years and this revealed a low hemoglobin concentration according to the references values of the participating laboratory (i.e. hemoglobin < 13.7 g/dL and < 12.1 g/dL for males and females, respectively), a further extensive laboratory assessment was performed. This assessment consisted of measuring hemoglobin, mean corpuscular volume (MCV), reticulocyte count, leukocyte count, thrombocyte count, lactate dehydrogenase, vitamin B12, folic acid, creatinine, ferritin, transferrin, and serum iron. These laboratory tests were chosen based on the Dutch guideline and tests regularly ordered by the participating internal medicine physicians [11]. The test results, together with the patient’s sex and age were stored anonymously in the database. As we wished to study anemia etiologies during diagnostic work-up, we excluded data of patients already known to have anemia in the previous 2 years.

This study was approved by the institutional review board of the Albert Schweitzer Hospital and was conducted in accordance with the Declaration of Helsinki.

### Definitions

We defined a set of 8 laboratory-orientated etiologies of anemia based on literature, the Dutch general practitioners’ guideline of anemia and the references values of the participating laboratory (Table 1) [3–6, 11–14]. These eight etiologies were ACD, renal anemia, IDA, hemoglobinopathy, suspected hemolysis, suspected bone marrow disease, vitamin B12 deficiency and folic acid deficiency. The use of these definitions ensures a uniform representation of the laboratory etiologies and takes into account the possibility of multiple etiologies in a patient. The strict application of the definitions made the combination of IDA and ACD impossible.

**Table 1:** Laboratory-orientated etiologies of anemia

	Definition
**Anemia of chronic disease**	Serum ferritin > 100 μg/L and at least one of the following transferrin ≤3.60 g/L or iron < 14 (male) / < 10 (female) μmol/L
**Renal anemia**	Estimated glomerular filtration rate < 45 mL/min/1.73m^2^
**Iron deficiency anemia**	Serum ferritin < 25 (male) or < 20 (female) μg/L
**Hemoglobinopathy**	Hemoglobin electrophoresis was conducted in case of low MCV (< 80 fl) in combination with increased erythrocyte count (> 6.2 (male) or > 5.4 (female) μl) (and followed by genetic testing).
**Suspected hemolysis**	Lactate dehydrogenase > 241 U/L and reticulocytes > 2.5%
**Suspected bone marrow disease**	Reticulocytes < 2.5% and leukocyte count < 4.3 or > 10 10^9^/L and thrombocyte count < 150 / > 390 10^9^/L
**Vitamin B12 deficiency**	Serum vitamin B12 < 130 pmol/L
**Folic acid deficiency**	Serum folic acid < 5 nmol/L
**Multiple causes**	Satisfied more than one of the above definitions
**Uncertain**	Satisfied none of the above definitions

To be able to visualize and analyze trends between anemia etiology and patient characteristics, we clustered some etiologies that had a low incidence. Thus, vitamin B12 deficiency and folic acid deficiency were taken together, and hemoglobinopathy, suspected hemolysis and suspected bone marrow disease were clustered as ‘other etiologies’. In addition, hemoglobin values were clustered into three groups proportionally. Severe anemia was defined as hemoglobin ≤9.7 g/dL; moderate anemia as hemoglobin > 9.7 and ≤ 12.9 g/dL for males and > 9.7 and ≤ 11.3 g/dL for females. Mild anemia was defined as hemoglobin > 12.9 and < 13.7 g/dL for males and > 11.3 and < 12.1 g/dL for females.

### Statistical analysis

Laboratory protocol violations had resulted in missing laboratory values (Table 1). To avoid selection bias as a consequence of the exclusion of patients with missing values, we applied single imputation using an expectation-maximization algorithm [15]. For nine laboratory values, the percentage of missing values was below one. For creatinine, however, it was 19.6%. This could be ascribed to the fact that GPs could follow two pathways when they requested laboratory analysis, one of which did not include creatinine. Still, this percentage was low enough to allow single imputation to be applied. Characteristics of the study population are described in terms of frequency, median and interquartile ranges. The distribution of underlying etiologies of anemia and the multiple aspect of the etiologies is described using counts and relative frequencies. We visualized the distribution of the etiologies of anemia by age, sex and level of anemia severity. To assess whether the etiology of anemia is associated with age, sex and/or the severity of anemia, we performed a multinomial logistic regression analysis. The dependent variable was anemia etiology (ACD, renal, IDA, vitamin B12 or folic acid deficiency, other, multiple etiologies or uncertain). The independent variables were age (continuous variable), sex (male/female) and anemia severity (mild/moderate/severe). Model assumptions were tested and met. We used SPSS for Windows, version 24 (IBM Corp., Armonk, NY, USA), for data handling and analyses. All statistical tests were two-sided and we considered a *p*-value *p* < 0.05 statistically significant.

## Results

### Inclusion and general characteristics

**Fig. 1:**
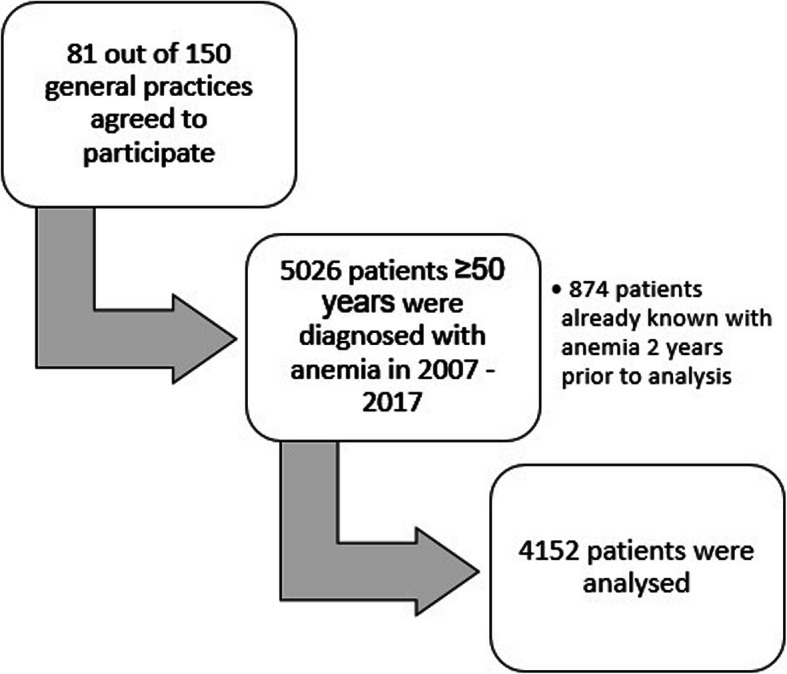
Consort diagram of the patient cohort

From February 1, 2007 until February 1, 2017, a total of 5026 patients from the collaboration project were registered with anemia in the laboratory system. A total of 874 patients (17%) already known with a recent history of anemia 2 years prior to laboratory analysis were excluded. Thus, data of 4152 patients with a new diagnosis of anemia were included in the analyses. A consort diagram shows the process of the selection of the patients (Fig. 1). The median age of this population was 75 years (IQR 64–83 years) and 2036 (49%) were male. A total of 887 patients (21%) had one or more missing laboratory values (Table 2).

**Table 2:** General characteristics of the study population (*n* = 4152)

	Median (interquartile ranges) / count (%)	Missing, counts (%)
**Age (per year)**	75 (64–83)	
**Male sex**	2036 (49%)	
**Hemoglobin (g/dL)**		–
**-Male**	12.9 (12.1–13.4)	
**-Female**	11.4 (10.6–11.8)	
**Reticulocytes (%)**	1.0 (0.8–1.4)	16 (0.4)
**Leukocyte count (10**^**9**^**/L)**	7.1 (5.7–9.0)	20 (0.5)
**Thrombocyte count (10**^**9/**^**L)**	269 (216–344)	28 (0.7)
**LDH (E/L)**	306 (221–372)	11 (0.3)
**eGFR (mL/min/1.73m**^**2**^**)**	> 60 (54 - > 60)	812 (19.6)
**Ferritin (μg/L)**		7 (0.2)
**-Male**	157 (60–321)	
**-Female**	81 (21–207)	
**Transferrin (g/L)**	2.38 (2.06–2.82)	10 (0.2)
**Serum iron (μmol/L)**		10 (0.2)
**-Male**	11.2 (6.5–15.6)	
**-Female**	8.8 (4.9–12.5)	
**Vitamin B12 (pmol/L)**	288 (209–430)	26 (0.6)
**Folic acid (nmol/L)**	16 (11–25)	38 (0.9)
**Uncertain anemia etiology**	819 (20)	
**Single anemia etiology**	2430 (59)	
**Multiple anemia etiologies**	903 (22)	
**-Two**	- 811 (90)	
**-Three**	- 88 (10)	
**-Four**	- 4 (0.4)	

### Anemia etiology

According to the definitions of laboratory etiologies of anemia, a single etiology was found in 2430 patients (59%); multiple etiologies in 903 patients (22%); and an uncertain etiology in 819 patients (20%). Among the patients with multiple etiologies of anemia, two etiology categories were assigned to 811 patients (90%); three to 88 patients (10%); and four to 4 patients (0.4%).

The most common etiology was ACD (54.5% of patients), followed by IDA (19.1% of patients) and renal anemia (13.8% of patients) (Table 3). IDA and ACD were the most frequent single etiologies of anemia, i.e. 82 and 68% of diagnoses, respectively. In contrast, folic acid deficiency and suspected bone marrow disease most often were part of multiple etiologies (94 and 88% of diagnoses, respectively) (Table 3).

**Table 3:** Frequency of underlying anemia etiologies

Underlying etiology	Single^**a**^ (***n*** = 2430)	Multiple^**a**^ (***n*** = 903)	Total^**b**^ (***n*** = 4152)
**Anemia of chronic disease**	1536 (68)	728 (32)	**2264 (54.5)**
**Renal anemia**	130 (23)	441 (77)	**571 (13.8)**
**Iron deficiency anemia**	646 (82)	146 (18)	**792 (19.1)**
**Hemoglobinopathy**	8 (35)	15 (65)	**23 (0.6)**
**Suspected hemolysis**	20 (17)	101 (83)	**121 (2.9)**
**Suspected bone marrow disease**	38 (12)	274 (88)	**312 (7.5)**
**Vitamin B12 deficiency**	49 (24)	153 (76)	**202 (4.9)**
**Folic acid deficiency**	3 (6)	44 (94)	**47 (1.1)**

^a^Count (percentage of total count of etiology), ^b^Count (percentage of total study cohort)

Among the 903 patients in whom multiple etiologies were found, the commonest combinations were ACD with renal anemia (*n* = 307, 34%), ACD with suspected bone marrow disease (*n* = 178, 20%) and ACD with vitamin B12 deficiency (*n* = 69, 8%).

### Anemia etiology in subgroups

**Fig. 2:**
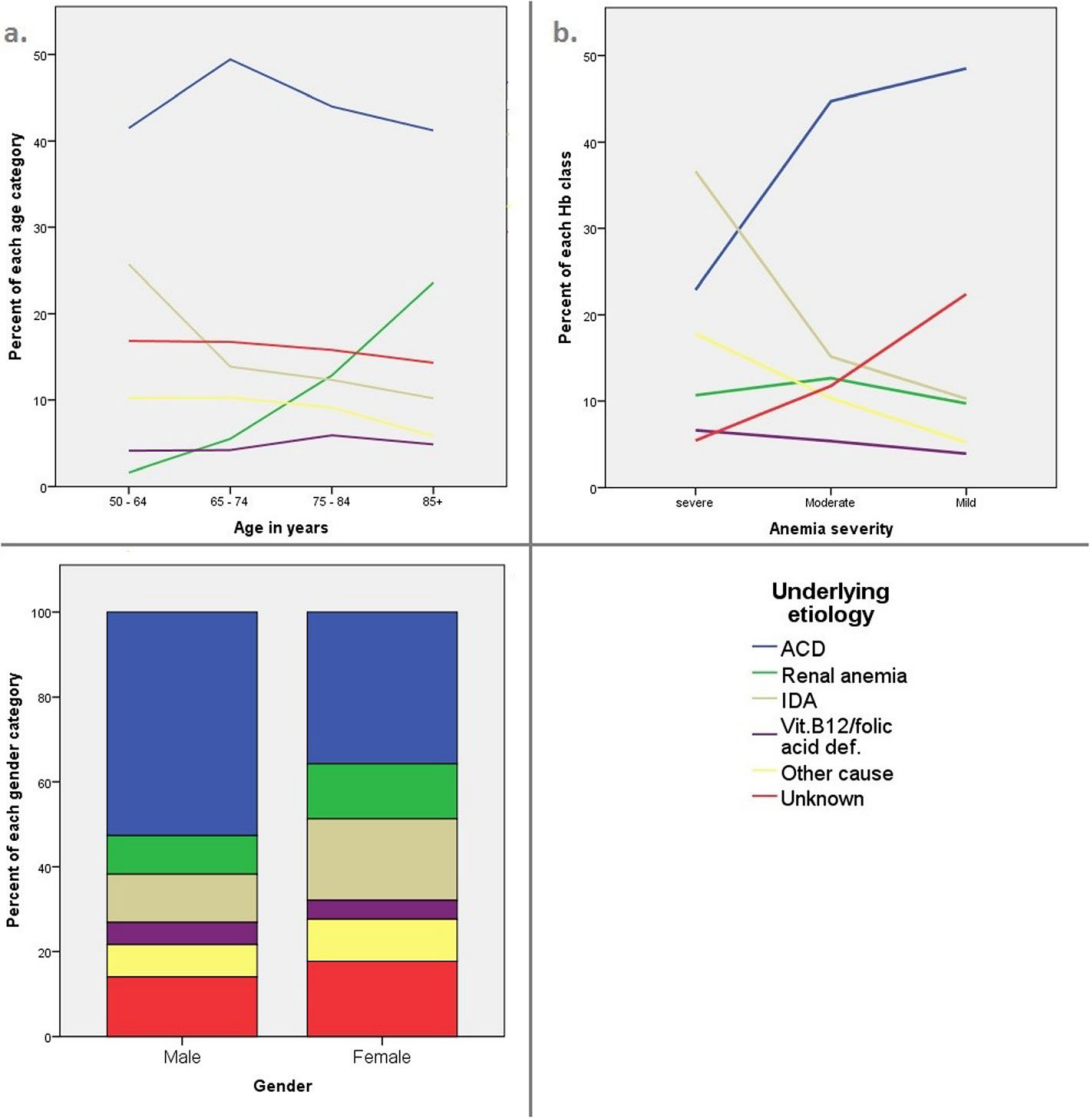
Distribution of patient characteristics among the anemia etiologies. Color legend. Blue: anemia of chronic disease. Green: renal anemia. Beige: iron deficiency anemia. Purple: vitamin B12/folic acid deficiency. Yellow: other cause. Red: unknown

The distribution of anemia etiologies in relation to specific patient characteristics is visualized in Fig. 2**.** Concerning age, the number of IDA etiologies declines with age, whereas the number of renal anemia cases increases with age (Fig. 2a). Regarding the severity of anemia, mainly ACD and uncertain etiology are more common in patients with mild anemia. Severe anemia is predominantly seen in relation with IDA (Fig. 2b). Looking at the sex distribution, we find that ACD etiology is more common in men, while IDA etiology is more common in women (Fig. 2c).

### Predictors of anemia etiology

A multinomial logistic regression was performed to assess whether the anemia etiology could be predicted from the patient’s age, sex and/or the severity of anemia. Anemia of uncertain etiology was defined as reference group. The results are shown in Table 4. Most striking was the association of ACD with male sex (OR 2.18, 95% CI 1.83–2.60), while IDA (OR 0.72, 95% CI 0.57–0.90) and other etiologies (OR 0.51, 95% CI 0.29–0.90) were more associated with female sex. Severe anemia was significantly associated with IDA (OR 13.12, 95% CI 8.63–19.94), other etiologies (OR 8.95, 95% CI 4.16–19.25) and the presence of multiple etiologies (OR 7.95, 95% CI 5.28–11.96).

**Table 4:** Multinominal logistic regression of anemia etiology

Anemia etiology	Median (range) or count (%)	OR (95% CI)	***P*** - value
**Unknown (*n*** **= 819)**	75 (50–99)	Reference group	
**-**Age (per year)	348 (42.5)		
**-**Male gender			
Anemia severity	587 (71.7)		
**-**Mild	200 (24.4)		
**-**Moderate	32 (3.9)		
**-**Severe	75 (50–99)		
**ACD (*n*** **= 1536)**			
**-**Age (per year)	73 (50–101)	1.00 (0.99–1.01)	0.65
**-**Male gender	959 (62)	2.18 (1.83–2.60)	< 0.001
Anemia severity			
**-**Mild	879 (57)		*Ref*
**-**Moderate	608 (40)	1.32 (1.10–1.59)	0.003
**-**Severe	49 (3)	0.98 (0.62–1.55)	0.92
**Renal (*n*** **= 130)**			
**-**Age (per year)	86 (63–102)	1.10 (1.07–1.12)	< 0.001
**-**Male gender	40 (30.8)	0.87 (0.57–1.31)	0.50
Anemia severity			
**-**Mild	78 (60.0)		*Ref*
**-**Moderate	40 (30.8)	1.58 (1.06–2.36)	0.03
**-**Severe	12 (9.2)	2.61 (1.27–5.38)	0.010
**IDA (*n*** **= 646)**			
**-**Age (per year)	67 (50–103)	0.96 (0.95–0.97)	< 0.001
**-**Male gender	237 (36.7)	0.72 (0.57–0.90)	0.003
Anemia severity			
**-**Mild	257 (39.8)		*Ref*
**-**Moderate	232 (35.9)	2.90 (2.29–3.68)	< 0.001
**-**Severe	157 (24.3)	13.12 (8.63–19.94)	< 0.001
**Vit B12 / FA def. (*n*** **= 52)**			
**-**Age (per year)	79 (50–100)	1.02 (1.00–1.05)	0.07
**-**Male gender	23 (44.2)	1.12 (0.63–2.00)	0.69
Anemia severity			
**-**Mild	34 (65.4)		*Ref*
**-**Moderate	18 (34.6)	1.32 (0.74–2.35)	0.35
**-**Severe	–	NA	NA
**Other etiologies (*n*** **= 66)**			
**-**Age (per year)	72 (50–100)	0.98 (0.96–1.00)	0.09
**-**Male gender	19 (28.8)	0.51 (0.29–0.90)	0.02
Anemia severity			
**-**Mild	30 (45.5)		*Ref*
**-**Moderate	23 (34.8)	2.68 (1.52–4.71)	< 0.001
**-**Severe	13 (19.7)	8.95 (4.16–19.25)	< 0.001
**Multiple (*n*** **= 903)**			
**-**Age (per year)	79 (50–103)	1.02 (1.01–1.03)	< 0.001
**-**Male gender	410 (45.4)	1.18 (0.97–1.44)	0.10
Anemia severity			
**-**Mild	356 (39.4)		*Ref*
**-**Moderate	397 (44.0)	2.94 (2.39–3.63)	< 0.001
**-**Severe	150 (16.6)	7.95 (5.28–11.96)	< 0.001

## Discussion

### Summary

Extensive laboratory analysis in the anemic patient from the general population enables to establish an etiology diagnosis more often. We found that 22% of patients have multiple etiologies defining their anemia. Moreover, many combinations of etiologies of anemia are possible. This study also estimated that age, sex and anemia severity are related to several anemia etiologies.

### Comparison with existing literature

We found that ACD, IDA and renal anemia were the most common etiologies of anemia among the studied population, which is in line with previous studies [3–6]. The etiology of the anemia was uncertain for 20% of patients, which proportion is much lower than the 26–44% reported in previous studies [3–6]. An explanation could be our inclusion of suspected bone marrow disease, hemoglobinopathy and suspected hemolysis as anemia etiologies, which was not done in previous studies [3–5]. Another explanation could be a lower incidence of uncertain anemia when a more regular extensive laboratory analysis is performed. This hypothesis is supported by a previous study in which only 8% of hospitalized anemia patients had an uncertain anemia etiology, most likely due to a more extensive laboratory analysis [9, 10]. Nevertheless, for most patients an anemia etiology can be diagnosed based on the laboratory tests results. This information is of great relevance for the general practitioners as it provides insight in the additional diagnostic work-up and prevents unnecessarily (hematology) referral.

To date, data on multiple etiologies of anemia has been scarce. In our study, a multiple etiology was found in 22% of patients from the general population. Especially vitamin B12 deficiency and folic acid deficiency were often part of a multiple anemia etiology. Therefore, extensive laboratory analysis might yield results that were not expected based on history and clinical presentation, but which may have treatment implications. Performing an extensive laboratory analysis in case of low hemoglobin concentrations is therefore recommended. This will reduce the number of missed causes of anemia and creates an optimal treatment framework. Furthermore, the laboratory protocol used for this study has shown to be effective at a minimal increase in costs [8]. In conclusion, an extensive laboratory analysis should be a standard first step during the diagnostic work-up of a newly diagnosed anemia patient, independently of the clinical presentation.

In this study, the distribution of anemia etiologies among specific patient characteristics showed a number of interesting trends. The peak of IDA etiology in the younger age group might be due to the arbitrary age cut-off of 50 years for inclusion. We offset this cut-off to exclude hypermenorrhea as predominant cause of IDA, but hypermenorrhea might still be present in some patients above 50 years. Furthermore, IDA is predominantly seen in patients with severe anemia, which might be explained by the more insidious course of anemia often seen in patients with IDA. As a consequence of the insidious course, the patient presents with symptoms at a later stage, resulting in a more severe anemia at moment of diagnosis. We also found that other etiologies presented more frequently with severe anemia. This finding highlights the relevance of additional tests (i.e. genetic screening or bone marrow biopsy) based on the clinical suspicion and family history. The mild anemia seen in patients with uncertain anemia etiology has been described in literature before [16]. Overall, Andres et al. noted no correlation between the severity of anemia and its underlying cause. We were able to demonstrate significant associations, which might be due to our large cohort resulting in a more precision of observed point estimates [10]. Regarding associations with the patients’ sex, we observed a trend towards twice as many ACD etiologies among men compared with women. Previous studies showed a broad range in this ratio [3, 5]. The trend we observed might have resulted from the strict definition of ACD including ferritin > 100 μg/L. The median ferritin level in the women in our study was lower than that in men (Table 2: 81 μg/L versus 157 μg/L, respectively). Therefore, women might be less likely to meet our definition of ACD.

### Strengths and limitations

A major strength of our study design is that it permitted to establish the onset of anemia. This was achieved by excluding patients already known with anemia 2 years previously. Another strength is the large cohort, which increased the precision of observed point estimates. Eighty-one out of 151 GP practices registered at our laboratory system participated in the study. These 81 GP practices can be considered reflective of all 151 GP practices in the area of Dordrecht. Therefore, we assume that there are no considerable differences between the distribution of anemia etiology among participating and non-participating GP practices.

As a limitation of our study, we did not know the indications for blood analysis; these are not registered. We, therefore, could not match a patient’s clinical information with the results of the blood analysis. We used a laboratory-orientated uniform enforcement when defining the etiologies and acknowledge the limitation of the missing clinical information. Still, our approach means a solid step forward in the anemia diagnostic work-up and we encourage the use of clinical information during the diagnostic process. On top of that, we are aware of the fact that the gold standard for anemia etiology diagnosis would be a bone marrow biopsy. However, this examination would be non-ethical to apply on each patient and can’t be performed in general practice setting.

We are aware of the fact that the WHO uses slightly different cut-off values of hemoglobin for anemia and not all laboratories have the same reference values. However, we decided to maintain the reference values according to the participating laboratory. A limitation is a gap in the analysis of ferritin values between 20/25–100 μg/L and vitamin B12 levels between 130 and 200 pmol/L, which is a consequence of our strict definitions. In the current study design, it was not possible to add additional parameters such as serum transferrin receptor, methylmalonic acid and homocysteine as additional parameters for further interpretation. This might have resulted in an underestimation of IDA, ACD and vitamin B12 deficiencies.

Although we defined a standard extensive laboratory protocol to be performed in each patient, data were missing for a proportion of patients. We applied single imputation to avoid selection bias and thus could include data of all patients in the analysis.

## Conclusions

In clinical practice, the extensive laboratory analysis during the diagnostic work-up of anemia patients seems to be valuable. Not only because more patients can be assigned an etiology, but also because the possible multiple aspect of etiologies can be effectively analyzed. Moreover, a patient’s age, sex and the severity of anemia may serve as markers that can guide the diagnostic work-up, resulting in a faster diagnosis and treatment initiation of the underling disease causing anemia.

## Data Availability

The dataset used and analysed during the current study are available from the corresponding author on reasonable request.

## Abbreviations

IDA: Iron deficiency anemia
ACD: Anemia of chronic disease
GP: General practitioner
MCV: Mean corpuscular volume
IQR: Interquartile range
